# Association of *ABCB1* (C3435T) and *ABCC1* (G2012T) Polymorphisms with Clinical Response to Atorvastatin in Iranian Patients with Primary Hyperlipidemia

**DOI:** 10.18869/acadpub.ibj.21.2.120

**Published:** 2017-03

**Authors:** Niusha Behdad, Javad Kojuri, Negar Azarpira, Amir Masoomi, Soha Namazi

**Affiliations:** 1 Department of Pharmacotherapy, Faculty of Pharmacy, Shiraz University of Medical Sciences, Shiraz, Iran; 2 Department of Cardiology, Faculty of Medicine, Shiraz University of Medical Sciences, Shiraz, Iran; 3 Transplant Research Center, Shiraz University of Medical Sciences, Shiraz, Iran

**Keywords:** Hypercholesterolemia, Statin, Gene polymorphisms

## Abstract

**Background:**

Atorvastatin is prescribed for the primary and the secondary prevention of coronary artery diseases. A wide variation in inter-individual statin response suggests that genetic differences may contribute to this variation. This study investigated the association of *ABCB1* (C3435T) and *ABCC1* (G2012T) polymorphisms with clinical response to atorvastatin in Iranian primary hyperlipidemic patients.

**Methods:**

Individuals (n=179) with primary hypercholesterolemia were enrolled, and peripheral blood samples were collected. Genotyping of two polymorphisms were performed by amplification refractory mutation system PCR.

**Results:**

Following four weeks of treatment, a significant reduction of LDL-C was observed in variant groups (CT+TT) of *ABCB1* (*P*=0.018) and wild-type group (GG) of *ABCC1* genes (*P*=0.029). Logistic regression analysis revealed a significant difference between male and female responses to 10 mg/day atorvastatin (*P*=0.004, odds ratio=0.2, CI 95%=0.06-0.6).

**Conclusion:**

Our finding indicated that these polymorphisms may be attributed to LDL-C serum levels in the primary hypercholesterolemia patients receiving atorvastatin.

## INTRODUCTION

Coronary artery disease (CAD) was responsible for approximately 300,000 deaths in 2010[1]. It has also been indicated to have a strong association with dyslipidemia[2]. The third report of the National Cholesterol Education Program[3] emphasizes the necessity of treatment of lipoprotein abnormalities in patients at high risk for coronary heart disease (CHD). The discovery of 3-hydroxy-3-methylglutaryl-coenzyme reductase inhibitors (statins) introduced a highly effective therapeutic approach for the reduction of both morbidity and mortality of patients with established CHD[4]. Serum lipid concentration and individual’s responsiveness to statin are influenced by environmental and genetic factors over 50%[5]. Pharmacogenetic studies have already identified more than 30 genes affecting statin.

P-glycoprotein (P-gp) and multidrug resistance protein 1 (*ABCC1*) play an important role in drug transport through the cell membrane, which may contribute to drug disposition and response[6]. P-gp is encoded by a polymorphic gene named multidrug resistance 1 (*ABCB1*), located on chromosome 7. Until now, more than 20 polymorphisms have been recognized; some of which have been associated with altered P-gp expression and activity[6-8].

Several investigations have focused on a synonymous polymorphism in the *ABCB1* gene, *C3435T* and reported that this polymorphism is associated with variation in atorvastatin efficacy and safety[9-11]. The *C3435T* (rs1045642) is located in the exon 26 and has a relationship with differences in total serum cholesterol, LDL-C, and HDL-C in response to atorvastatin in American populations[12]. *ABCC1* appears to be ubiquitously expressed in many human tissues and transports a wide spectrum of substrates and toxicants[13]. A non-synonymous *G2012T* (rs 45511401) polymorphism, located in exon 16, is common among Caucasian populations[14]. Recently, one study has found an association between polymorphisms in *ABCB1* and *ABCC1* genes and differences in serum LDL-C and HDL-C levels, which indicates the potential role of these polymorphisms in the pharmacogenetics of the atorvastatin[4,15]. Regarding these points, this study, for the first time, examined the association of *ABCB1* (*C3435T*) and *ABCC1* (*G2012T*) polymorphisms with para-clinical and clinical responses to atorvastatin in Iranian patients with hyperlipidimia.

## MATERIALS AND METHODS

### Study population

Patients with primary hypercholesterolemia were selected from the outpatients, who were evaluated for the presence of CAD, at the cardiovascular disease clinics affiliated with Shiraz University of medical sciences (Shiraz, Iran) between January 2012 and October 2013. The study protocol was approved by the Ethics Committees of Shiraz University of Medical Sciences (code number 91-01-36-5018). Pregnant women as well as individuals with serum triglycerides above 400 mg/dl or with thyroid disease (TSH≥5 or ≤0.4 µu/dl), renal failure (SCr≥2 mg/dl for women, SCr≥2.5 mg/dl for men)[16], liver diseases (ALT≥40 mg/dl), diabetes mellitus (who had two consecutive FBS≥126 mg/dl) and individuals treated with oral contraceptives and lipid-lowering drugs were not enrolled in the study[16]. All the patients signed an informed consent form. Four weeks before the study, all the subjects visited a dietitian and were instructed to consume a low-cholesterol diet (total daily fat intake 25 to 35% of total calories, trans fats to less than 7% of calories)[16]. Information on age, gender, body mass index, blood pressure or anti-hypertensive drug history, physical activity (20 minutes walking per day)[16], family history and past medical history of each patient were recorded. Alcoholic and smoker patients were excluded from the study due to the limited numbers. Individuals were categorized into CHD and non-CHD groups according to National Cholesterol Education Program ATP-III guidelines[3]. For non-CHD subjects, the risk factor was calculated, and global risk factor assessment was carried out to determine the initial treatment goals. All cases had two or more risk factors and were treated with 10, 20 or 40 mg atorvastatin (Sobhan Darou, Tehran, Iran) orally once daily for four weeks. The patients received an appropriate dose of atorvastatin according to their ideal LDL-C goal (patients with CHD, LDL-C≤70 mg/dl and LDL-C≤130 mg/dl for non-CHD patients).

### Lipid measurement

One week before and four weeks after atorvastatin administration, peripheral blood samples were collected from the individuals after an overnight fasting (8-12 h)[16]. Total serum cholesterol, HDL-C, LDL-C and triglyceride concentrations were measured. Before and after the administration of atrovastatin, ALT and creatinine kinase were determined to detect the possible adverse drug reactions.

### Genotyping

Genomic DNA was extracted from buffy coat of EDTA-treated peripheral blood samples by the DNP kit (CinnaGen, Tehran, Iran) according to the manufacturer’s instruction. Genotyping of the *C3435T* polymorphism on *ABCB1* and *G2012T* on *ABCC1* was carried out using amplification refractory mutation system PCR method. Briefly, PCR assays were performed with 30-40 ng genomic DNA, 10 µl Master mix (0.1 U/µl DNA polymerase, 32 mM (NH_4_)_2_SO_4_, 5.5 mM MgCl_2_, 130 mM Tris-HCl, pH 8.8, 0.4 mM of each dNTP, 0.02% Tween-20), 0.6 µM *ABCB1* reveres primer (5’-GGA GAC CCC CTT ATA AATC-3’), 1 µM *ABCB1* forward primer (5’-TGG TGT CAC AGG AAG ATA TC-3’), 1 µM *ABCC1* reveres primer (5’-CCA CCA CGG CCA CCA AAT CAA-3’) and 0.7 µM *ABCC1* forward primer (5’-CTG CCT CAC TTC AAG GGA CAC-3’). The thermal cycle (Techno, Genius, UK) protocol for *ABCB1* and *ABCC1* consisted of an initial denaturation at 95ºC for 5 min, followed by 30 and 35 cycles of denaturation at 94ºC for 25 s and 40 s, annealing at 59 ºC for 35 s and 62ºC for 45 s and extension at 72ºC for 25 s and 40 s. PCR products were analyzed by 2% agarose gel electrophoresis and stained by SYBR Green dye.

### Statistical analysis

Continuous variables were presented as mean±SD. Categorical variables were reported as counts (percentage). To find any potential association between genotypes in candidate genes and demographic phenotypes and response to atorvastatin, we first divided the patients into two different dosage groups, individuals treated with 10 mg atorvastatin per day and those who were treated with 20 or 40 mg. To evaluate the association between *ABCB1* and *ABCC1* polymorphisms and serum lipid concentration, individuals carrying the homozygous form of the less common allele were grouped with the heterozygous carriers (variant group), and those carrying the homozygous form of the more common allele were put in the wild-type group. General linear regression model was employed to evaluate the association of *C3435T* and *G2012T* genotypes with LDL-C reduction. Logistic regression analysis was carried out to compare the effect of demographic factors and *C3435T* and *G2012T* polymorphisms on clinical response to atorvastatin (or achievement of LDL-C to therapeutic goal). The allele frequency of these variants was assessed for deviation from the Hardy-Weinberg equilibrium using Chi-square test. A *P*<0.05 was considered statistically significant. SPSS 17 software (SPSS Inc., Chicago, Illinois) was used for statistical analysis.

## RESULTS

### Subjects’ characteristics and the frequency distribution of the polymorphisms

Patients (n=179) with primary hypercholesterolemia were enrolled in this study. Based on the laboratory data, the mean±SD of LDL-C, triglyceride, total cholesterol and HDL-C were139.8±27.2, 163.4±60.8, 217.7±37.9 and 42.6±8.6 mg/dl, respectively. As shown in Table 1, environmental factors have similar frequencies between the two dosage groups (*P*>0.05). The genotype and allele distribution of two polymorphisms are shown in Table 1. The success rate for obtaining the LDL-C level was 76%.

**Table 1:** Clinical characteristics, genotype and allele frequency of *ABCB1*/*ABCC1* in patients with hyperlipidemia

Characteristics	Total (n=179)	First group (n=104)	Second group (n=75)	*P* value
Age (mean±SD, year)	58.9±9.4	59.4±8.7	58.3±10.1	0.3
BMI^a^ (mean±SD, kg/m^2^)	24.0±3.4	23.7±3.4	24.4±3.3	0.96
Female no.(%)	66(36.9)	42(40.4)	24(32.0)	0.07
Male no.(%)	113(63.1)	62(59.6)	51(68.0)	0.08
Positive family history no.(%)	83(46.4)	39(37.5)	44(58.7)	0.24
Positive physical activity^b^ no.(%)	139(77.7)	81(77.8)	58(77.3)	0.95
Positive diet control^c^ no.(%)	145(81.0)	87(83.7)	58(77.3)	0.05
SNP/genotype				
*ABCB1* (C3435T) no.(%)				
CC	5(2.8)	2(1.9)	3(4)	0.07
CT+TT	174(97.2)	102(98.1)	72(96.0)	
T allele	195(54.4)	115(55.3)	80(53.3)	0.8
C allele	163(45.6)	93(44.7)	70(46.7)	
*ABCC1* (G2012T) no.(%)				
GG	106(59.2)	61(58.7)	45(60.0)	0.8
GT+TT	73(40.8)	43(41.3)	30(40.0)	
G allele	262(73.2)	155(74.5)	107(71.3)	0.08
T allele	96(26.8)	53(25.5)	43(28.7)	

First group received atorvastatin 10 mg/day and the second group atorvastatin 20 and 40 mg/day.

a Body mass index;

b 20-min walking per day;

c Consumption of low fat and calories and high-fiber diet per day

### Effect of *ABCC1* and *ABCB1* polymorphisms on serum lipid level

The percentage change in lipid parameters by genotype is shown in Table 2. Multivariate logistic regression analysis showed significant differences between males and females’ responses to atorvastatin following four weeks of treatment with 10 mg/day atorvastatin (*P*=0.004, odds ratio=0.2, CI 95%= 0.06-0.6). The result indicated that women are better responders to the therapy by five-fold. Furthermore, no significant association was detected between the *ABCB1* (*C3435T)* and *ABCC1* (*G2012T)* polymorphisms and clinical response with different dosages of atorvastatin in Iranian hyperlipidemic patients. Linkage disequilibrium between *C3435T* and *G2012T* polymorphisms was not observed in our study population (*P*=0.56).

**Table 2:** Percentage change in lipid parameters based on C3435T genotype in *ABCB1* and G2012T in *ABCC1* in each dosage group after four weeks of atorvastatin therapy

Variables	*ABCC1*		*P* value	*ABCB1*		*P* value
	
	GG	GT+TT		CC	CT+TT	
First group^a^						
LDL-C	-37±11	-33±13	0.02	-23±10	-35±12	0.01
HDL-C	6±2	8±1	0.11	7±3	8±3	0.73
Triglyceride	-17±10	-18±12	0.82	-10±6	-8±8	0.22
Total cholesterol	-26±12	-23±13	0.06	-22±10	-24±11	0.26
						
Second group^b^						
LDL-C	-43±12	-43±11	0.81	-44±11	-43±11	0.63
HDL-C	6±4	7±2	0.26	7±5	7±4	0.85
Triglyceride	-17±8	-20±4	0.16	-20±10	-18±9	0.55
Total cholesterol	-30±15	-29±13	0.29	-30±14	-29±13	0.53

a n=104; atorvastatin 10 mg/day);

b n=75; atorvastatin 20 and 40 mg/day; LDL-C, low density lipoproteins-cholesterol; HDL-C, high density lipoproteins cholesterol (the mean percentage change±SD, mg/dl)

## DISCUSSION

The present investigation showed that the frequencies of the *ABCB1* genotype (CC: 2.80%, TT: 11.70% and CT: 85.50%) were not similar to those found in Iranian population in previous studies (*P*>0.05)[17-19]. Azarpira *et al*.[17] have found *ABCB1* wild-type genotype (*3435CC*) in 19% of Iranian renal transplant patients, whereas 51% and 30% were heterozygous (*3435CT*) and homozygous (*3435 TT*), respectively. The genetic risk variants such as *APOE, PCSK9, CDKN2A* and *CDKN2B* are associated with conventional risk factors for CAD and very commonly occur in 50% of the population[20]. In this light, we suggest that *ABCB1* polymorphism might be considered as a genetic risk factor for CAD development, and it may affect the genotype profile of these patients. However, no data were found regarding *ABCB1* genotype distribution in healthy control group to compare with our patient group.

*De novo* sequencing at *ABCC1* was performed on 142 healthy individuals from four different populations (Chinese, Malay, Indian and Caucasian) to identify polymorphisms at multidrug resistance protein 1/*ABCC1*[21]. T allele frequencies in Caucasians (2.8%), Indians (1.4%), Chinese (0%) and Malay (0%) were not similar to those found in our study (T; 25.5%)[21]. Furthermore, *G2012T* polymorphism was common among Caucasians but it was not detected in Asian populations[21].

In the present study, logistic regression analysis revealed significant differences between males and females’ response to atorvastatin regarding the achievement of LDL-C goal after four weeks of treatment with 10 mg/day atorvastatin (*P*=0.004, odds ratio=0.2, CI95%=0.06-0.6). Women in the first group showed better response than men. One study indicated lower mean area under the concentration-time curve and shorter half-life of atorvastatin in women than in men[22]. In a previous study on Japanese women with primary hypercholesterolemia LDL-C level was decreased to a greater extent in women than men after three months of treatment with atorvastatin[23], which was in accordance with our findings. The current study revealed that four weeks of treatment with 10 mg/day atorvastatin significantly reduced LDL-C level in various groups of *ABCB1* gene polymorphism (CT+TT) and wild-type group of *ABCC1* gene (GG). The LDL-C reduction demonstrated that the effect of these polymorphisms on atorvastatin efficacy was more clear in low-dose atorvastatin. Taking dose dependency of atorvastatin into consideration[16], we suggest that in higher doses of atorvastatin, the effect of dose is more significant than the polymorphism effects.

In a recent study, Kajinami *et al*.[10] found no association between CC genotype of *ABCB1* and smaller reductions in LDL-C, but larger increases were observed in HDL-C after treatment with atorvastatin (10 mg/day). Furthermore, our study confirmed the investigation by Hoenig *et al*.[24] on Australian patients treated with 80 mg atorvastatin. The result indicated that the CC genotype at the *C3435T* polymorphism in *ABCB1* was associated with reduced atorvastatin efficacy regardless of the cholesterol metabolism following a six-week administration of atorvastatin. In Alzoubi *et al*.[25] study, both the TT genotype of *G2677T (ABCB1)* and the TT genotype of the *C3435T (ABCB1)* polymorphisms were associated with lower levels of LDL-C after atorvastatin treatment in Jordanians hypercholesterolemic patients. However, the effects of atorvastatin on the levels of total cholesterol, triglyceride and HDL-C had no relationship with any of the genotypes in both polymorphisms. In Rebecchi *et al*.[15] study on Brazilian population, no relationship was found between LDL-C reduction and *ABCC1* (*G2012T)* polymorphism, but *ABCB1* and *ABCC1* mRNA levels were modulated by atorvastatin and *ABCB1* *G2677T/A* polymorphism. Also, *ABCB1* baseline expression was related to the differences in the serum LDL-C and ApoB in response to atorvastatin. Unequal group sizes and the risk of selection bias with the groups are the main limitations of this study. A short-term follow-up and the lack of control group for comparison may be considered as other important limitations. However, we concluded that in a low dose of atorvastatin, *ABCB1* and *ABCC1* polymorphisms may have an effective impact on atorvastatin efficacy in reduction of LDL-C serum level in Iranian patients with primary hypercholesterolemia.
